# Bipolar vs. unipolar scaling in dynamic network analyses of Ecological Momentary Assessment data

**DOI:** 10.1371/journal.pone.0314102

**Published:** 2025-03-18

**Authors:** Arwin Nemani, Bettina Hufschmidt, Viktoria Kohl, Lucie Sendig, Mareike Ebert, Desiree Bonarius, Stefan G. Hofmann, Ulrich Stangier

**Affiliations:** 1 Department of Clinical Psychology and Psychotherapy, Goethe University Frankfurt, Frankfurt am Main, Germany; 2 Department of Psychology, Philipps-University Marburg, Marburg, Germany; University of Catania, ITALY

## Abstract

This study examined the statistical and clinical benefits of using bipolar versus unipolar scaling in dynamic network analysis of Ecological Momentary Assessment (EMA) data. Methods: Forty-seven students completed EMA reports three times daily for five weeks via either unipolar (n = 24) or bipolar (n = 23) scales. The data were analyzed to construct idiographic network models. Results: The bipolar scaling group presented significantly lower zero inflation (2.37% vs. 10.31%, U = 2407756, r = 0.75, p < .05) and greater response variability. Network analysis revealed more participants with significant network edges in the bipolar group (69.57% vs. 41.67%, χ²(1) = 12.06, p = .0007). Additionally, the bipolar group had lower odds of zero responses than the unipolar group did (p = .038). Conclusion: Bipolar scaling enhances EMA data quality by reducing zero inflation and increasing variability, resulting in richer dynamic network models. Further research is needed to confirm these findings in clinical populations.

## Introduction

Ecological momentary assessment (EMA) is a pioneering method that enables real-time capture of psychological states and behaviours, offering detailed insights into the dynamic interplay of emotions, cognitions, and behaviours [1]. An important tool within the EMA is the visual analog scale (VAS), a reliable and validated instrument for measuring subjective experiences, particularly in real-time contexts. VAS has been extensively employed in both medical and psychological research because of its sensitivity to subtle changes in psychological states and its high temporal precision [2].

In psychology research, EMAs facilitate the collection of intensive longitudinal data, playing a key role in the dynamic network approach to understanding psychological processes [3]. The VAS is particularly valued for its flexibility, as noted by McCormack et al. [4], making it adaptable to various research contexts, including the measurement of complex psychological states. This adaptability is crucial when tools such as bipolar scaling are employed to capture a more nuanced and comprehensive representation of psychological processes. This approach contrasts sharply with traditional diagnostic models, which often view mental disorders as static, latent entities that manifest as symptoms. Dynamic network models, on the other hand, conceptualize psychological issues as systems of functionally interrelated elements [5]. Establishing causality within these models requires highly frequent, repeated measurements of variables to assess their temporal relationships [6].

High-frequency data collection is essential for accurately capturing the temporal fluctuations and interdependencies of psychological processes [7]. Research suggests that at least 50 to 100 measurement points are necessary to reliably estimate dynamic network models [8]. Such detailed data collection reduces the risk of spurious connections and enhances the reliability of the identified networks. However, small sample sizes can adversely affect the accuracy of model estimates [9].

A significant challenge in dynamic network models is the frequent occurrence of zero inflation, where data entries often result in zeros, indicating a lack of variance and, consequently, connectivity within the models [10,11]. This issue can arise from insufficient data, erroneous inputs, or an exclusive focus on negative events and maladaptive processes, leading to sparse networks that fail to capture the true complexity of psychological states[5,12]. Addressing zero inflation is essential to accurately reflect the sparsity and heavy-tailed edge count distributions observed in empirical data, thereby preventing biased models and enhancing the representation of complex system dynamics [8,10]. A potential reason for this issue is the use of unipolar scales that focus solely on negative events and maladaptive processes. Previous studies have demonstrated that such scales reduce data variability, hindering the identification of significant edges in dynamic network models [13].

Moreover, frequent responses to symptom assessments in clinical trials can negatively affect mood. While some evidence suggests that intense self-monitoring can increase awareness of negative responses and contribute to greater self-control [14,15], there is also some evidence for pronounced individual differences in reactivity to daily assessments of negative mood [e.g., 16,17].

In this context, the current study investigates the statistical and practical implications of utilizing bipolar versus unipolar scaling in EMAs. Bipolar scales, which encompass both positive and negative poles, may offer a more nuanced and comprehensive representation of complex interdependent processes than unipolar scales, which capture only a single pole—either positive or negative [18]. It is hypothesized that bipolar scales increase sensitivity in detecting emotional and cognitive states, thus enhancing the validity of the collected data [19]. The circumplex model of affect, which inherently uses bipolar scales to capture the full spectrum of emotional experiences, supports this approach by facilitating a more holistic understanding of emotional dynamics [20]. Larsen and Diener [21] further endorse bipolar scaling, noting that it enables a more comprehensive assessment of emotional and cognitive states by capturing both positive and negative dimensions.

By employing bipolar scaling, researchers can better capture the bidirectional nature of emotional and cognitive processes, which is essential for modeling the intricate interplay of these factors in mental health. Additionally, many psychological instruments include both negatively and positively formulated items [22–26]. The present research aims to address these limitations by exploring whether bipolar scaling, compared with unipolar scaling, enhances data variability and improves the modeling of dynamic networks. Enhanced data variability is particularly important for reducing zero inflation, ensuring that dynamic models are rich and informative [10,27]. Additionally, the use of bipolar scaling may increase participant engagement and acceptance of EMA, as it allows for the assessment of both positive and negative aspects of experiences, leading to more balanced and comprehensive self-reflection [28]. The versatility of the VAS in various research contexts, as discussed by Müller et al. [4], underscores its potential application in dynamic network analysis.

Therefore, this study seeks to empirically validate preliminary network models by hypothesizing that bipolar scaling provides a more differentiated representation of the complex interdependencies among psychological processes than unipolar scaling does. We anticipated that judgments of psychological dimensions on bipolar scales would lead to a reduced number of zero responses and an increased number of significant edges in dynamic network models. Additionally, we explored the effects of bipolar scaling versus unipolar scaling on symptoms of psychological distress, including anxiety and depression, hypothesizing that bipolar scaling is associated with significantly lower levels of psychological distress than unipolar scaling is.

## Method

### Participants

The recruitment for this study took place between October 10, 2023, and November 31, 2023. The study included 47 participants, all of whom were students recruited from Goethe University Frankfurt via digital flyers. Participants were selected based on their willingness to participate in an intensive Ecological Momentary Assessment (EMA) study. The mean age of the participants was 25 years (SD = 5), ranged from 21 to 40 years with a predominantly female sample (73.60%). Regarding educational background, 65.22% of the participants held a university degree, 30.43% had a high school diploma, and 4.35% had successfully obtained an advanced technical college certificate. Additionally, 56.52% of the participants reported having a part-time job alongside their university studies.

Each participant received compensation of 175 EUR after completing the study. Participants were recruited through advertisements that emphasized the benefits of gaining a better understanding of situations where they feel unwell, improving their self-observation skills, and earning money. Interested individuals were instructed to scan a QR code for more information and to participate in a prescreening process. The prescreening process included a brief online questionnaire assessing eligibility criteria such as current student status, age (minimum 18 years), ability to use a smartphone for Ecological Momentary Assessment (EMA) reporting, and willingness to provide informed consent.

Participants were randomly assigned to one of two groups based on the scaling method used in their EMA: the unipolar group (n = 24) reported their emotional and cognitive states via unipolar scales, while the bipolar group (n = 23) used bipolar scales for the same assessments. The assignment was not based on specific prescreening criteria, as the aim was to ensure an equal distribution of participants across both groups and to allow for natural variability within each group. Randomization was conducted to avoid potential biases and to maintain group comparability for the intended comparisons.

### Ethic statement

All participants provided written informed consent prior to their inclusion in the study. Only adults (18 years and older) were eligible to participate, ensuring that all individuals had the legal capacity to consent. The consent process involved a thorough explanation of the study’s objectives, procedures, and any potential risks, both verbally and in written form. Participants were given ample time to review the information and ask questions before signing the written consent form. No verbal consent was used; hence no additional documentation or witness procedures were required.

All procedures involving human participants were performed in accordance with the ethical standards of the institution’s Human Research Ethics Committee of the Faculty of Psychology and Sports Science at the Johann-Wolfgang-Goethe University Frankfurt (Reference No.: 2023-62). The Ethics Committee confirmed that the study conforms to ethical standards, and no particular problems were anticipated.

### Procedure

The participants were given a questionnaire by mail to help them identify the situational context of a psychological problem and specify maladaptive processes involved (emotions, body sensations, cognitions, cognitive processing, behaviours, motivation). In the group with bipolar scaling, the participants were also instructed to specify adaptive processes as counterparts. If problems or questions occurred, they were clarified with the study organizer via e-mail. The items defined in this way were used to create the EMA items.

The participants were then instructed to complete EMA reports three times a day over a period of five weeks, resulting in approximately 100 measurement points per participant. Each EMA report consisted of six questions designed to capture real-time fluctuations in affect, cognition, behaviour, contextual factors, processing, and motivational factors. Data collection was facilitated by a mobile app, which prompted participants at random times within predefined windows to ensure representative sampling of their daily experiences. The app also logged the time and date of each response to ensure compliance and tracked the temporal sequence of reported states and events. Before starting the EMA phase, the participants were asked what they thought was their individual central process, which had the greatest impact on the other processes.

### Measures

The primary measures used in the study included the Ecological Momentary Assessment (EMA), the Depression Anxiety Stress Scales (DASS-21) [29], and evaluation items on the EMA.

The EMA involved participants using a mobile application to report their current emotional and cognitive states, behaviours, and contextual factors multiple times daily, with items including both unipolar and bipolar scales depending on group assignment. The DASS-21, a 21-item self-report questionnaire developed by [29], was used to assess levels of depression, anxiety, and stress at the beginning and end of the study period.

Additionally, participants were asked to evaluate their experience with the EMA process, including perceived burden, impact on self-awareness and the perceived fit of the model with one’s own inner processes, as well as the perceived usability of the model to initiate change.

### Data analysis

The data analysis aimed to construct idiographic network models for time series data via ecological momentary assessment (EMA) measurements. The R package graphicalVAR was employed to generate temporal networks on the basis of single-lag models. These networks were designed to analyze interactions between variable data over time [8]. The key parameters considered in the analyses included lambda and the penalty parameter. Lambda ensures the precise capture of network connections between variables [8], whereas the penalty parameter optimizes the network structure and enhances the robustness of our results [30].

The network estimation was conducted via graphical VAR functions to determine the strength of connections between variables in the network. The focus was on temporal networks with single-lag models to appropriately capture temporal dependencies. Additionally, detrending analysis via the R package forecast highlighted short-term fluctuations and minimized long-term trends [31].

The interpretation of the obtained insights was directly related to our specific research questions and adhered to the methodological principles of network analysis. We used the centrality of outstrength or outdegree as a crucial measure to assess the importance of individual variables in terms of their interactions within the network.

Data analysis focused on comparing the outcomes between the unipolar and bipolar scaling groups. Statistical methods included Mann‒Whitney U tests to evaluate differences in zero inflation rates and F tests and Levene’s tests to assess the variability and distribution of responses between the two groups. The primary outcome measures were the frequency of zero responses, the variability of reported states, and the structure of dynamic networks derived from the EMA data.

Hierarchical logistic regression was performed to investigate the likelihood of reporting at least one “0” response among various psychological measures (situation, emotion, cognition, behaviour, processing, body response, motivation) between two groups: unipolar and bipolar. The dependent variable was dichotomized such that a value of 1 indicated the presence of at least one “0” response among the measures, and 0 otherwise. The independent variable was group membership (1 = unipolar, 2 = bipolar). Given the hierarchical structure of the data, with repeated measures nested within individuals over days and multiple observations per day, random intercepts for subjects were included to account for intraindividual variability. This model allowed us to control for the nested nature of the data and appropriately estimate the effects of group membership on the likelihood of experiencing a “0” response.

This methodological approach aimed to determine the effectiveness of bipolar scaling versus unipolar scaling in capturing the complexity of psychological processes in real time. By carefully controlling for these factors, this study sought to provide insights into the applicability of these scaling methods for process-based therapeutic interventions, ultimately aiming to improve the accuracy and richness of the data used to inform therapeutic strategies.

## Results

### Zero inflation and data variability

**Fig 1:**
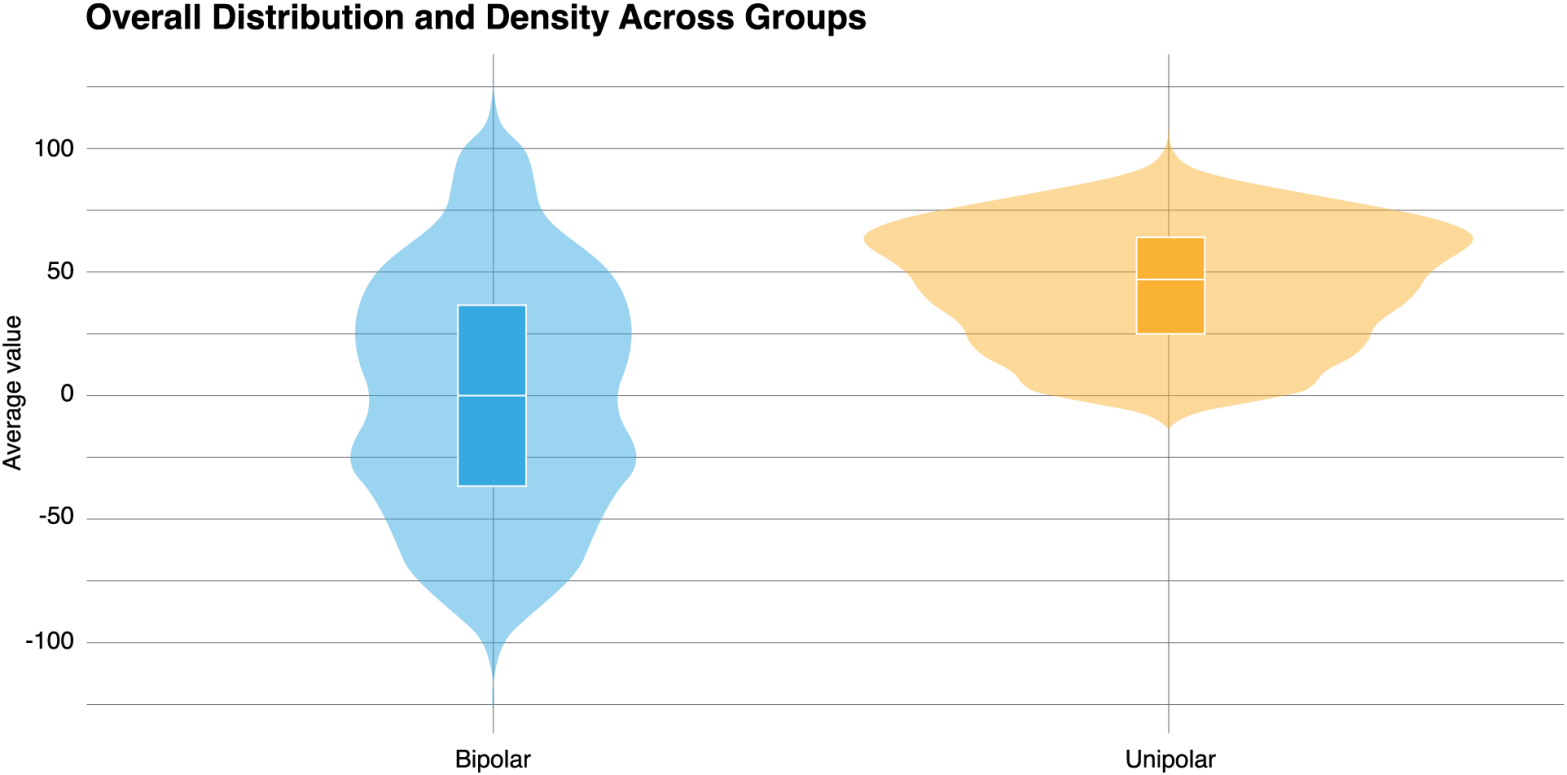
Overall distribution and density across groups.

The analysis revealed significant differences in zero inflation between the unipolar and bipolar scaling groups. The percentage of zero responses (2.37%) was significantly lower in the bipolar scaling group than in the unipolar scaling group (10.31%), as indicated by the Mann‒Whitney U test (U = 2407756, r = 0.75, p < .05). This reduction in zero responses suggests that bipolar scaling enhances the sensitivity of EMA in capturing a broader range of emotional and cognitive states. The distribution of responses is illustrated in Fig 1.

The hierarchical logistic regression model revealed several key findings: the intercept was 0.219 (p = 0.0008), indicating the baseline log odds of reporting a “0” response in the unipolar group. This translates to a probability of approximately 55.4%, suggesting a substantial baseline likelihood of reporting a “0” response in this group. The effect of group membership had a coefficient of −0.140 (p = 0.038), indicating that the bipolar group had lower log odds of reporting a “0” response than the unipolar group did. This significant difference suggests that the bipolar group is less likely to report a “0” response. The random effects revealed a group variance of 0.041, indicating some variability between subjects within the groups. However, both the day and measurement variances were negligible, suggesting minimal variability within days and between measurements within a day.

Furthermore, the data variability was assessed via F tests and Levene’s tests. The results indicated that the bipolar group exhibited significantly greater variability in their responses across multiple dimensions, including emotion, cognition, behaviour, and contextual factors. Levene’s tests revealed significant differences in the variances between the groups, with the bipolar group demonstrating a more distributed data set. This increased variability suggests that bipolar scaling captures a wider spectrum of psychological states, providing a richer dataset for dynamic network analysis.

### Dynamic network analysis

Dynamic network models were constructed to explore the interdependencies among psychological processes. The chi-square test for independence revealed a significant difference in the distribution of networks with and without connectivity between the bipolar and unipolar groups (χ²(1) = 12.06, p = .0007). In the bipolar group, a greater proportion of participants had dynamic networks with significant edges (69.57%) than did the unipolar group (41.67%). These findings are summarized in Table 1, which displays the distribution of networks with and without edges in the unipolar and bipolar groups. This finding indicates that bipolar scaling improves the identification of significant interactions within the network models.

**Table 1:** Distribution of dynamic networks with edges in the unipolar and bipolar groups.

Group	Networks with Edges	Networks without Edges	Total
**Bipolar (23)**	16 (69.57%)	6 (30.43%)	23
**Unipolar (24)**	10 (41.67%)	14 (58,33%)	24
**Total**	26	20	46

“Networks with edges” refers to participants whose dynamic network models show interactions between psychological processes.

The analysis of network centrality further supported the benefits of bipolar scaling. The participants in the bipolar scaling group had more central nodes with high outstrength centrality, suggesting that key psychological processes were more effectively identified and targeted in these networks.

### Evaluation of EMA experience

The participants’ evaluations of their EMA experience were analyzed. There were no significant differences between the groups in terms of perceived burden, awareness of inner processes, or the impact of answering the questions on awareness. Additionally, there were no significant differences in how awareness made participants feel worse or better. Both groups reported that their model accurately described their inner processes and that they could use the model to improve their mental health. Overall, both groups found the EMA process manageable and insightful.

The participants were asked before the EMA survey what they thought their central node (the central process that most influences the others) was, which was compared to the results in the individual networks. For 20% of the participants, the empirical result matched the assumed result.

### Impact on psychological well-being

The impact of EMA on participants’ psychological well-being was assessed via the DASS-21. Paired t tests comparing pre- and poststudy scores revealed no significant differences in depression between the unipolar group (pre: M = 6.95, SD = 4.33; post: M = 7.86, SD = 5.25) or the bipolar group (pre: M = 5.91, SD = 4.35; post: M = 7.85, SD = 5.67).

Similarly, there were no significant differences in anxiety between the unipolar group (pre: M = 5.29, SD = 4.67; post: M = 6.67, SD = 4.23) or the bipolar group (pre: M = 4.39, SD = 3.43; post: M = 4.75, SD = 3.84).

The stress levels also did not differ significantly from pre- to postexposure for the unipolar group (pre: M = 8.57, SD = 4.72; post: M = 10.00, SD = 3.82) or the bipolar group (pre: M = 7.43, SD = 4.32; post: M = 8.50, SD = 4.68).

This suggests that while EMA, particularly with bipolar scaling, enhances data quality and network modeling, it does not directly influence short-term psychological well-being, as measured by the DASS-21.

The descriptive statistics for the Depression Anxiety and Stress Scales (DASS-21) before and after the study are presented in Table 2.

**Table 2:** Descriptive statistics for Depression, Anxiety, and Stress Scales (DASS-21) pre- and postintervention.

	Unipolar	bipolar
**Pre M (SD)**		
Anxiety	5.29 (SD = 4.67)	4.39 (SD = 3.43)
Depression	6.95 (SD = 4.33)	5.91 (SD = 4.35)
Stress	8.57 (SD = 4.72)	7.43 (SD = 4.32)
**Post M (SD)**		
Anxiety	6.67 (SD = 4.23)	4.75 (SD = 3.84)
Depression	7.86 (SD = 5.25	7.85 (SD = 5.67)
Stress	10 (SD = 3.82)	8.5 (SD = 4.68)

M = Mean, SD = Standard Deviation.

## Discussion

The present study aimed to explore the statistical and practical implications of utilizing bipolar versus unipolar scaling in ecological momentary assessment (EMA) within the context of improving dynamic network models in psychology research. The findings provide substantial evidence that bipolar scaling, when used with the visual analog scale (VAS), offers significant advantages over unipolar scaling in capturing the complexity of psychological processes.

One of the most compelling findings was the significant reduction in zero inflation with bipolar VAS scaling. This reduction suggests that bipolar VAS scales are more sensitive and can capture a broader range of emotional and cognitive states. This aligns with the theoretical benefits of bipolar scaling discussed in the introduction, which proposed that bipolar VAS could offer a more nuanced and comprehensive representation of complex interdependent processes [19,20]. The enhanced data variability observed in the bipolar VAS group further supports this notion, indicating that bipolar VAS scales are better suited for capturing the dynamic nature of psychological processes, which is crucial for accurate dynamic network modeling [21].

Dynamic network analysis revealed that bipolar VAS scaling significantly improved the identification of significant edges within psychological networks. This finding is particularly relevant for understanding the role of psychological flexibility and cognitive-affective processes in dynamic psychological systems, as discussed by Westhoff et al. [32]. Their research emphasized the importance of these processes in adapting to changing psychological and environmental conditions, which aligns with our findings that highlight the utility of bipolar VAS scaling in capturing such complex interactions. Sung et al. [22] also support the use of bipolar scaling, noting that it captures a more comprehensive spectrum of emotional and cognitive states by considering both positive and negative aspects.

By identifying more central nodes and significant interactions, bipolar VAS scaling facilitates a more precise understanding of the key psychological processes that maintain maladaptive networks. This, in turn, can inform more targeted and effective therapeutic interventions. The reliability of the VAS, as demonstrated by Bijur et al. [2], supports its use in capturing rapid changes in psychological states, making it a robust tool for identifying central processes within dynamic networks.

Moreover, the statistical methods suggested by Heller et al. [33] underscore the importance of appropriately analyzing VAS data. They recommend using continuous ordinal regression or distribution-free methods, especially when dealing with the ordinal nature of VAS measurements. These methods ensure that the rich data collected through bipolar VAS scaling are accurately interpreted, enhancing the validity of the dynamic network models developed in this study.

The participants’ evaluations of their EMA experience indicated that the process was generally well received, with no significant differences in perceived burden between the unipolar and bipolar VAS groups. This suggests that despite the more complex nature of bipolar VAS scales, participants did not find them more burdensome to use. Moreover, the slightly higher level of satisfaction with self-reflection reported by the bipolar group suggests that bipolar VAS scales might enhance the perceived utility of EMA. This aligns with the notion that capturing both positive and negative aspects of experiences can lead to more balanced and comprehensive self-reflection, enhancing participant engagement and the overall effectiveness of the EMA process [29].

However, bipolar scaling may introduce complexity for participants unfamiliar with the concept of bipolar continuums, which assume opposite poles on a single continuum. This assumption could result in confusion when psychological states do not align neatly along a single axis, such as when participants experience high levels of both positive and negative emotions simultaneously. Future research could explore ways to enhance participant comprehension of bipolar scales, such as through additional instructions or training, to ensure consistent and accurate responses.

While the study revealed no significant short-term impact on psychological well-being as measured by the DASS-21, this does not negate the potential long-term benefits of using bipolar VAS scaling in EMAs. The primary goal of EMA in this context is to provide rich, real-time data that can inform process-based therapeutic interventions. Although the immediate psychological benefits might not be apparent, the enhanced data quality and improved network modeling facilitated by bipolar VAS scaling can lead to more effective long-term therapeutic outcomes by enabling more precise and individualized treatment planning.

The insights gained from bipolar VAS scaling are particularly relevant for process-based therapy (PBT), as described by Stangier et al. [34]. PBT emphasizes the importance of identifying and targeting the underlying psychological processes that contribute to an individual’s distress. By accurately mapping the dynamic interactions between these processes, bipolar VAS scaling can help clinicians pinpoint central nodes within maladaptive networks. This precision allows for more targeted interventions that can disrupt dysfunctional patterns and promote adaptive functioning. The clarity provided by bipolar VAS scaling enhances the therapist’s ability to tailor interventions to the specific needs of the patient, thereby improving therapeutic outcomes.

The study also revealed that most participants were not able to correctly predict the central process in their model but evaluated the model as accurately describing their inner processes and as useful for initiating change. This finding indicates that the EMA phase has led to an improvement in the perception of interactions within the individual network model, as most participants were convinced that EMAs had corrected their expectations in a subjectively correct direction.

The findings of this study have significant implications for the practical use of dynamic network models as tools that map psychological processes and can be used to initiate change. By providing a more nuanced and comprehensive representation of psychological processes, bipolar VAS scaling can enhance the accuracy and richness of the data used to inform therapeutic strategies. This aligns with the process-based approach’s emphasis on tailoring interventions to the specific interactive processes that contribute to an individual’s psychological distress [35]. The ability to identify central nodes and significant interactions within dynamic networks is essential for developing targeted interventions that can disrupt maladaptive patterns and promote adaptive functioning, which can increase the effectiveness of psychotherapy [36].

## Limitations

However, several limitations should be noted. The sample size of the present study was relatively small, and it was not a clinical sample, which limits the generalizability of the findings. Future research should include larger and diverse clinical populations to validate these results. Additionally, the short-term nature of this study did not allow us to observe potential long-term impacts of bipolar scaling on psychological well-being. Future longitudinal studies may explore whether bipolar scaling in EMA has a cumulative impact on mental health outcomes over time, especially in therapeutic settings. While bipolar scaling has been shown to reduce zero inflation and increase data variability, it is important to recognize that this method assumes a linear relationship between the poles of the scale [37].

This assumption may not always hold true in real-world settings. For example, an individual might report high levels of both rumination and mindfulness simultaneously, reflecting a complex interplay of psychological processes that are not easily captured by a single bipolar scale. These scenarios highlight the potential limitations of bipolar scaling in accurately representing multifaceted psychological states.

Furthermore, bipolar scales may introduce complexity that could confuse participants or lead to inconsistent responses. Ensuring that participants understand the nature of bipolar scales and can use them accurately is crucial for obtaining reliable data. Training and continuous support for participants may help mitigate this issue.

## Conclusion

In conclusion, while this study demonstrates the advantages of bipolar scaling in EMAs for capturing the complexity of psychological processes and enhancing data quality, it also underscores the need for further research to address the limitations associated with this method. Future studies should explore the long-term impact of bipolar scaling on therapeutic outcomes, include larger and clinical samples, and investigate ways to refine bipolar scales to better capture the nuances of psychological states. These efforts will help optimize the use of EMA in psychological interventions and improve the precision and effectiveness of personalized psychological treatments.
